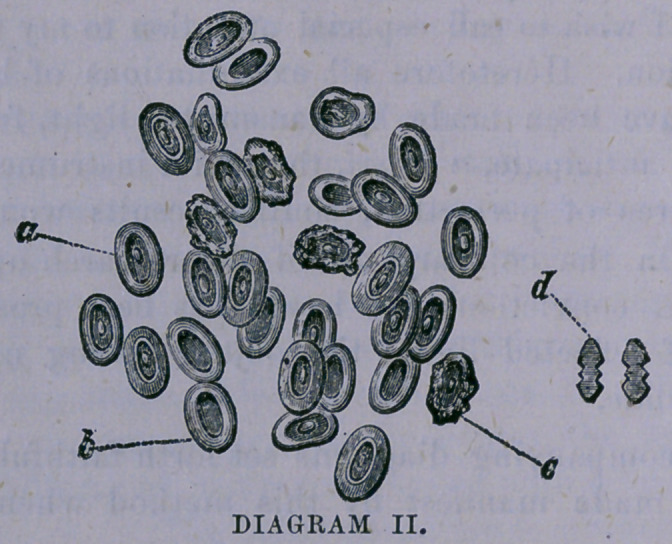# Discovery of a New Anatomical Feature in Human Blood Corpuscles

**Published:** 1869-04-15

**Authors:** J. W. Freer

**Affiliations:** Prof. of Physiology and Microscopic Anatomy, Rush Medical College


### CHICAGO MEDICAL JOURNAL.

VOL. XXVI.—APRIL 15, 1869.—No. 8.

### COMMUNICATIONS.

### *Discovery of a New Anatomical Feature in Human Blood Corpuscles.*

BY J. W. FREER, M.D., PROF. OF PHYSIOLOGY AND MICROSCOPIC ANATOMY, RUSH MEDICAL COLLEGE.

ARTICLE NO. I.

In the CHICAGO MEDICAL JOURNAL of May 15, 1868, Vol. XXV., No. 10, in a brief, hastily written article, I announced that I had, as I believed, discovered that human blood corpuscles "are not, as heretofore supposed, simply bi-concave disks; but, on the contrary, there may be seen, (by the use of Wale's Illuminator,) a nipple-like eminence in the center of the concavity of each well-formed disc. This papillary eminence is abont .00,000,1 of an inch in diameter at its base. That it is a true anatomical form, and not a change incidental to dessication, etc., is shown by its appearance at the instant of withdrawal of any given specimen, while the corpuscles are still plump and smooth in all other respects."

Continued investigation on this important subject has served only to confirm the announcement then made; and having had the opportunity of exhibiting corpuscles as herein described, to several eminent men of acknowledged scientific ability, and having received their opinion in corroboration of my own, I propose to set forth in this paper the views heretofore entertained upon this subject, and in contradistinction present my own more fully, illustrated by accompanying diagrams, which were drawn by an artist who had never before seen blood corpuscles, and, consequently to be taken as unprejudiced testimony.

Among physiologists and microscopic anatomists there has been but one generally-expressed opinion as set forth in standard works, illustrated and otherwise; *to wit*, that the human blood corpuscle is non-nucleated.

It may appear incautious, and even rash, to array one's self against evidence so weighty, but my convictions have ripened under careful and persistent iuvestigation, and I deem the announcement timely and worthy of earnest attention.

It may be superfluous to quote the opinions of authors with whom nearly all are familiar, but for a concise presentation of the subject I deem it advisable.

Todd and Bowman, in treating "of the structure of the red corpuscle" say: "The structure of the red corpuscle of most of the vertebrata may be readily demonstrated in the reptilia—that of the frog, for instance. It is distinctly a nucleated cell—consisting of a delicate cell membrane, within which is a granular nucleus, which may be rendered more distinctly granular by acetic acid. The nucleus is globular, and much smaller than the cell, and the interval between the inner surface of the latter and the outer surface of the former is filled by fluid which holds the coloring matter in solution. Corpuscles of this kind floated in pure water become distended by the endosmosis of it, burst, and give exit to their nuclei, while the shreds of the cell-membrane are scattered in the fluid."

"It cannot be shown, satisfactorily, that the bi-concave, circular corpuscle of human blood and of that of mammalia is of the same structure as this, because it cannot be demonstrated to consist of cell and nucleus. If it be as the blood-corpuscles of birds, reptiles and fishes undoubtedly are, a nucleated cell, the obscurity of its nucleus is probably due to one of two causes; either it is so large as accurately to fill the cell, leaving no space between the outer surface of the one and the inner surface of the other, or it is so extremely minute as completely to elude our means of observation."

"Mr. Wharton Jones supposes that the mammalian red corpuscle is a nucleus, the cell of which has existed only in the earlier stages of development. Kölliker, on the other hand, affirms that the nucleous disappears while the cell wall is persistent. All that microscopic examination with the highest powers and the best instruments shows respecting the structure of this corpuscle is, that it consists of a delicate membrane, enclosing a semi-fluid material, impregnated with the proper coloring matter of the blood; and that this structure may truly be assigned to it is amply proved by the change of form which it undergoes by the endosmosis of pure water, which will cause it to burst, and evacuate its contents, consisting of nothing more than some minute granules, none of which can be compared to a nucleus. So far as microscopic analysis would enable us to decide this question, we should be disposed to declare in favor of Mr. Jones' view; but it seems greatly opposed by two facts; first, that in the corpuscle of the lower vertebrata, the coloring matter is contained between the nucleus and the cell-wall, whereas, in the mammalian corpuscle it would be contained in the nucleus; and, secondly, that this peculiarity of structure is limited to one class of vertebrate animals. It receives support, however, from observing the several steps of the development, for the corpuscle exhibits a stage in which a nucleus is visible, (the stage of colored, nucleated cell,) and this nucleus, in the very large corpuscle of the elephant, and likewise in the very small corpuscle of the goat, exhibits a strict correspondence in size with the perfectly formed blood-corpuscles. But here, again, we notice the difficulty above referred to, that in this stage of nucleated cell, the color is found between the cell and the nucleus. It seems to us that further research is required, in order to determine the exact homology of the mammalian red corpuscle.

Mueller says: "The blood globules, both of the human and other mammiferæ ordinarily do not present the appearance of being nucleated, notwithstanding the generality of the presence of a nucleus in other classes render it probable that it also exists here. I believe, even, that by means of certain light I have sometimes been able to discover it in man. On treating human blood with dilute acetic acid the corpuscles suddenly disappear, and nothing remains but a few small granules, in regard to which one is in doubt as to whether they are or are not the nuclei of the globules."

Kölliker, in treating of the blood says: "The red blood-globules, more minutely examined, present the following characters:

Their form is usually that of a biconcave or plane, orbicular disc, with rounded borders, and, consequently, they present a different aspect to the observer, according as the surfaces or borders are turned towards him. In the former case they are pale-yellow, orbicular corpuscles, in which, according to the focussing of the microscopes, the slight central depression which always exists, is indicated, sometimes by an opaque spot in the center, the latter appearance admitting of being confounded with a *nucleus*. * * * * * The blood corpuscle is *constituted* of a very delicate, but nevertheless tolerably firm, and at the same time, elastic, colorless cell-membrane, composed of a protein substance closely allied to *fibrin*, and of colored viscid contents formed of *globulin* and hæmatin, which, in the adult, present no trace of morphological particles or granules, or of a *cell-nucleus;* they are, consequently, vesicles, whence the name 'blood-cells' is to be preferred."

Longet says: "with man and other mammiferous adults the blood globules appear to be deprived of nuclei. There is not even an exception in the family camelidæ, which, by the form of their globules, approach to those in which the presence of nuclei is not doubtful. That which has been taken for a nucleus is but the thin, central part of the disc. This remark already made by Hodgkin and Lister, has been confirmed by Henle, Donné, Wharton Jones, etc., notwithstanding various Micrographers admit that sometimes among the ordinary globules without nuclei one meets with those that appear nucleated. Such, at least, is the result of the observations made by Wharton Jones, Schultz, Nasse and Busk with man and other mammifera."

As all investigators are so nearly in harmony on the subject of the anatomy of these organic forms, the methods of observation having been uniformly the same, it is deemed unnecessary to make further quotations.

In presenting my own views in contradistinction to the foregoing I wish to call especial attention to my method of investigation. Heretofore all examinations of blood corpuscles have been made by transmitted light, from which we might anticipate, *a priori*, that, with instruments of the same degree of perfection, similar results would be obtained. On the contrary, all of the research upon which my present convictions are based has been prosecuted by the use of reflected light; the object resting upou a polished jet slide.*

The accompanying diagrams set forth faithfully the appearance made manifest by this method when properly pursued.

DIAGRAM I.

Diagram I.—*a*, represents the perfect, human blood corpuscle. It will be seen that the center presents a slight elevation, surrounded by an annular depression, while the circumference of the disc is, compatatively, thick, smooth and rounded.

*b*, represents a disc with serrated and shriveled margin, with a perfect central elevation remaining.

*c*, a corpuscle without the central elevation.

*d*, is a hypothetical diagram of a disc placed upon its edge.

DIAGRAM II.

Diagram II.—*a*, represents the perfectly formed, oval and characteristic disc, or globule, of the frog. In the central portion may be seen an oval depression, in the center of which can be observed the elevation representing the nucleus. The margin is shown to be rounded, smooth and thickened.

*b*, is like the former in every respect except the central papilla is absent.

*c*, a shriveled corpuscle.

*d*, a corpuscle as it would appear set upon its edge.

It will be seen from the foregoing explanation that these bodies do not all possess a nucleus, or central elevation. In fact, there are in both the human blood and that of the frog a large proportion, perfect in every other respect, that do not possess this peculiarity. Whether this fact is due to an absence of this feature on one of the surfaces of the corpuscle or to its non-existence in a certain per centage of cases, I am undetermined.

I have, as yet, examined but few pathological specimens. In one instance, however, in a case of advanced Typhoid fever, the corpuscles, without exception, were found serrated, shriveled and distorted in the most remarkable manner, with the exception of the nucleated center. This seemed to maintain its integrity.

The reader, by reference to the diagrams, will observe that the corpuscles, both of the human and reptilian blood, are analogous in their general features, as, witness the elevated nuclei in the centers, the surrounding depression and the elevated margins, together with the entire absence of nuclei in nearly equal proportions of each. It will also be observed that certain numbers are imperfect in outline, as if affected by disintegration; and this is true of a similar proportion of every specimen that I have ever examined. This apparent analogy of form and feature between mammalian and reptilian corpuscles only supports what is generally acknowledged should obtain with these bodies in order to bring them in harmony with the known homology of other tissues, from whatever species of animal obtained. I believe that it is never denied that nerve, muscular, bony and other tissues, when compared with their kind, are essentially alike in their anatomical constitution. Why should blood tissue form an exception to this general rule? Do not the corpuscles perform the very same function, whether floating in the blood of a reptile or in the vessels of man?

With one exception I have not, as yet, extended my observations to the condition of blood discs in the various parts of the body. In the spleen pulp I found them mainly imperfect in form, being generally serrated, shriveled, and seldom nucleated. Hæmatine, in granulated masses, was seen interspersed among the corpuscles. White corpuscles, in great numbers, were arranged around the margin of the specimen.

The Illuminator does not reveal any thing unusual concerning the white corpuscles; they merely appear as non-nucleated, globular bodies. It is remarkable, however, that they never associate themselves with the red discs, there seeming to be a perfect antagonism of position.

---

*NOTE.—Those who wish to repeat these observations, by means of Wale's Illuminator, will find it indispensable that the object-glass be arranged for uncovered objects, and the discs spread thinly over the surface of the slide. Corpuscles found in defibrinated blood are the best for observation. Those found in rouleaux do not show the nuclei satisfactorily.

## Figures and Tables

**DIAGRAM I. f1:**
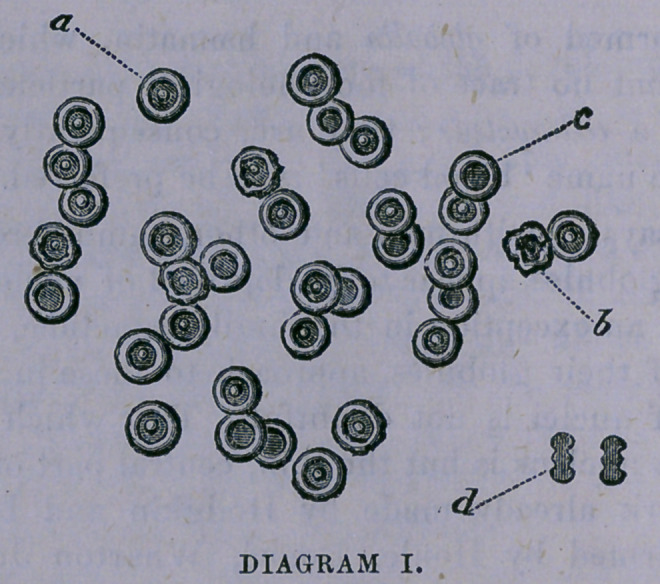


**DIAGRAM II. f2:**